# New validated HPLC methodology for the determination of (−)-*trans*-paroxetine and its enantiomer in pharmaceutical formulations with use of ovomucoid chiral stationary phase

**DOI:** 10.1007/s00216-013-7565-y

**Published:** 2014-01-10

**Authors:** Małgorzata Lisowska-Kuźmicz, Małgorzata Kantor-Boruta, Anna Jończyk, Małgorzata Jarończyk, Agnieszka Ocios-Bębenek, Aleksander P. Mazurek, Zdzisław Chilmonczyk, Maciej Jarosz

**Affiliations:** 1 National Medicines Institute, 30/34 Chełmska St, 00-725 Warsaw, Poland; 2 Faculty of Pharmacy, Medical University of Warsaw, 1 Banacha St, 02-097 Warsaw, Poland; 3 Faculty of Chemistry, Warsaw University of Technology, 3 Noakowskiego St, 00-664 Warsaw, Poland

**Keywords:** Chiral analysis, HPLC, Ovomucoid stationary phase, Pharmaceuticals, Paroxetine enantiomers

## Abstract

A new chromatographic method for the enantioseparation and the determination of (−)-*trans*-paroxetine and (+)-*trans*-paroxetine has been developed with the aid of amylose ovomucoid-based chiral stationary phase. The method is faster and five times more sensitive than procedures recommended previously: limit of detection and limit of quantification are 5 and 16 ng/mL, respectively [modified (Ferretti et al. in J Chromatogr B 710:157–164, 1998): 20 and 60 ng/mL]. It was carefully validated and applied for the determination of (−)-*trans*-paroxetine and (+)-*trans*-paroxetine in Parogen (Mc Dermott Laboratories Ltd.) and Xetanor (Actavis) coated tablets.

**Electronic supplementary material:**

The online version of this article (doi:10.1007/s00216-013-7565-y) contains supplementary material, which is available to authorized users.

**Figure:**
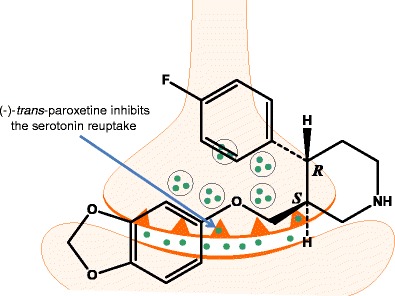
ᅟ

## Introduction

In case of chiral drugs, substantial differences in transport, distribution, metabolism, and excretion of particular enantiomers may be observed. Hence, for medicinal products marketed as formulations containing only one enantiomer, a very important issue is the content of the second enantiomer, which in the best case may appear as an inactive impurity but may also be responsible for undesired effects. The most popular techniques used for drugs enantiomeric purity estimation are high-performance liquid chromatography (HPLC) [1–3] and capillary electrophoresis [4, 5], enabling both enantiodifferentiation and enantiomers quantification.

Paroxetine ((−)-*trans*-paroxetine: (3S-trans)-(−)-(3S,4R)-4-(4-fluorophenyl)-3-[[(3,4-methylenedioxy)phenoxy]-methyl]piperidine) is a compound belonging to selective serotonin reuptake inhibitors, drugs used in major depression [6], obsessive-compulsive, as well as generalized and social anxiety disorders [3]. With the exception of a low affinity to muscarinic receptors, which is not relevant for therapeutic effects, it does not interact directly with monoamine neurotransmitter receptors [7]. Paroxetine, containing two chiral carbon centers may form four optical isomers—two for *cis*- and two for *trans*-paroxetine. In therapeutics, it is used as (−)-*trans*-paroxetine. In pharmaceutical formulations, its most likely not active enantiomer, (+)-*trans*-paroxetine, may also be present [2].

For the determination of paroxetine enantiomers, few HPLC stationary phases (including optically active metal complexes, cyclodextrin, penicillamine, and Pirkle type complexes) [3, 8] were examined, but reasonable enantioseparation was obtained only with the aid of amylose [3, 9, 10]- and cellulose [3]-based chiral stationary phases. Carboxymethyl-β-cyclodextrin was also recommended as mobile phase modifier improving efficiency of the process [11].

In the present paper, we describe new high-performance liquid chromatographic method for paroxetine enantiomers separation and determination with use of silica-based chiral stationary phase containing glycoprotein (ovomucoid [12, 13]) as chiral selector, more sensitive and offering better resolution than those recommended before. The method was validated and applied to paroxetine enantiomers quantification in two pharmaceutical formulations: ParoGen (Mc Dermott Laboratories Ltd.)- and Xetanor (Actavis)-coated tablets containing 20 mg paroxetine (as paroxetine hydrochloride, 22.22 mg). Validated HPLC method with amylose tris(3,5-dimethylphenylcarbamate) chiral stationary phase was used a reference procedure. It should be noted that the present method was developed as a result of extensive studies with such chiral selectors as hydrocarbons (cellulose and amylose), proteins, and cyklodextins. More than 20 aqueous and non-aqueous mobile phases were examined.

## Materials and methods

### Medicinal products

ParoGen-coated tablets, 20 mg paroxetine as paroxetine hydrochloride (22.22 mg; McDermott Laboratories Ltd.); Xetanor-coated tablets, 20 mg paroxetine as paroxetine hydrochloride (22.22 mg; Actavis).

### Reference substances (standards)

Paroxetine hydrochloride (anhydrous) ((−)-t*rans*-paroxetine hydrochloride) 96.2 % (Matrix Laboratories Ltd., WS batch number QC-3/PRH/WS001/008), quality assurance laboratory label 678a/PS/J; (+)-*trans*-Paroxetine hydrochloride (hemihydrate; Gedeon Richter Ltd. batch number R4B0310), quality assurance laboratory label 97a/PS/J; (+)-*trans*-Paroxetine hydrochloride (USP Paroxetine Related Compound C) 99.9 % (Sumika batch number STS-01), quality assurance laboratory label 97/PS/J.

Placebo of medicinal product ParoGen-coated tablets (Alphapharm batch number P576), quality assurance laboratory label 687a/PS/J; placebo of medicinal product Xetanor, prepared according to manufacturer recipe.

### Reagents

*n*-Hexane 95 % and acetonitryle HPLC (Labscan Ltd.), ethanol and methanol HPLC (Merck), ethanolamine (2-aminoethanol reagent plus ≥99 %; Sigma-Aldrich), potassium phosphate, phosphoric acid, potassium hydroxide (POCh).

### Apparatus

Chromatographic set-up Dionex Ultimate 3000 LC composed of LC pump, UV-VIS detector, autosampler, thermostat, and Chromeleon software. Chiral separations were performed using:

- ovomucoid (glycoprotein) immobilized on aminopropylsilane-derivatized silica (Ultron ES-OVM 150 × 4.6 mm, 5 μm, Agilent Technologies) column-mobile phase: 10 mM phosphate buffer pH 3.5 (1.36 g K_2_HPO_4_/1,000 ml water, pH adjusted to 3.5 with H_3_PO_4conc._ or 1 M KOH)-acetonitrile (98:2, *v*/*v*), detection UV (295 nm, *t* = 23 °C), flow rate (1.5 mL/min). For the preparation of calibration curves, methanol-pH 3.5 phosphate buffer solutions containing 0.01 to 100 μg/mL of the analyte were applied (injection volumes, 20 μL)
- silica based amylose tris(3,5-dimethylphenyl)carbamate (Chiralpak AD-H 250 × 4.6 mm, 5 μm, Daicel Chemical Industries Ltd.) column (modified procedure described elsewhere [9])-mobile phase: hexane-ethanol-ethanolamine (80:20:0.2, *v*/*v*/*v*), flow rate (1.0 mL/min), detector UV (295 nm, *t* = 23 °C). For the preparation of calibration curves, methanol-ethanol solutions containing 0.01 to 100 μg/mL of the analyte were applied (injection volumes, 20 μL).

### Methods

#### Analysis of medicinal preparations ParoGen 20 mg-coated tablets and Xetanor 20 mg-coated tablets

Paroxetine standard solution preparation: 2 mg of (−)-*trans*-paroxetine standard was weighed and transferred into 100 mL volumetric flask. Then, 10 mL of methanol was added and the flask was filled with: ethanol for the analysis with use Chiralpak AD-H column, 10 mM pH 3.5 phosphate buffer for the analysis with use of Ultron ES-OVM column.

Placebo extract preparation: accurate weight amount of placebo corresponding to excipients content in a tablet (280 mg) was placed into 100 mL volumetric flask and 10 mL methanol was added. The sample was sonicated for ∼10 min. The volumetric flask was filled with methanol and the resulted suspension was filtered. 5.0 mL of the filtrate was placed into 50 mL volumetric flask and filled with appropriate solvent or solution (depending on the column used, see above).

Extraction of paroxetine from ParGen and Xetanor pharmaceutical formulations: tablets of the formulations were carefully ground. Accurate weight amount of each preparation corresponding to one dose (300 mg) was placed into 100 mL volumetric flask and extracts were prepared as described in above para.

Determination of analyte recovery within the content range of 80–120 % (according to ICH Validation of Analytical Procedures [14]): accurate weight amounts of placebo corresponding to excipients contained in one tablet (280 mg) were transferred into nine volumetric flasks (100 mL). Accurately weighed amounts of (−)-*trans*-paroxetine hydrochloride were poured into each flask: *ca*. 16 mg (80 % dose) to first three flasks, *ca*. 20 mg (100 % dose) to subsequent three flasks and *ca*. 24 mg (120 % dose) into the last three flasks. Ten milliliter methanol was added to each flask. The flasks were sonicated for ∼10 min, filled with methanol and the resulted suspensions were filtered. Of each filtrate, 5.0 mL was placed into 50 mL volumetric flask and filled with ethanol (Chiralpak) or phosphate buffer (Ultron).

## Results and discussion

### Robustness of the method

**Fig. 1:**
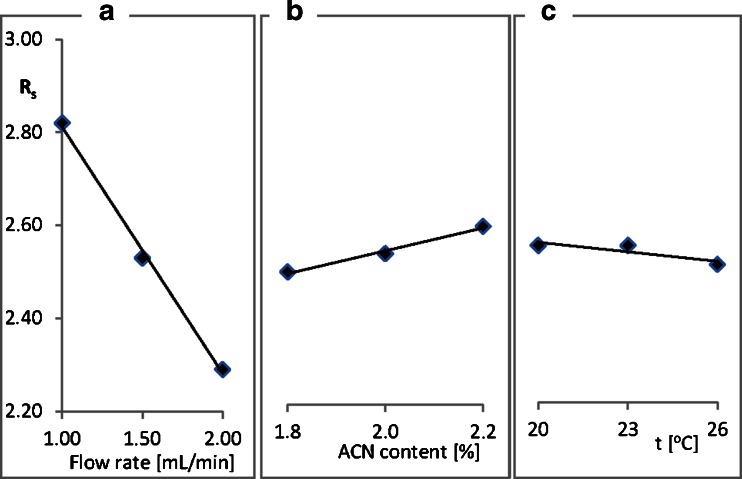
Influence of flow rate (**a**), acetonitrile content (**b**), and temperature (**c**) on the resolution of (±)-paroxetine on ovomucoid stationary phase

Influence of mobile phase composition and flow rate as well as column temperature on the precision of retention times and peak areas measurements and selectivity was examined. Twenty-microliter portions of (−)-*trans*-paroxetine hydrochloride standard solution (in phosphate buffer) were injected onto a column and chromatograms were registered (*n* = 6 for each measurement). Precision of retention times (*t*_*R*_), peak areas measurements and resolution (*R*_*s*_) were calculated. The obtained results prove good repeatability of the developed method with relative standard deviation (RSD) for retention times and peak areas between 0.11 and 3.09 %. It was also found, that changing acetonitrile content (±0.2 %), column temperature (±3 °C) and flow rate (±0.5 mL/min) did not much influence resolution factor (|Δ*R*_*s*_| < 0.3) although with the flow rate elevation decrease in the resolution factor could be observed (Fig. 1).

For the estimation of intraday precision (repeatability) of the method, eight 20-μL portions of the (−)-*trans*-paroxetine hydrochloride standard solution were injected onto column within the same day in six series. Precision of measurements of peak area (RSD = 0.29 %; confidence level, *p* < 0.05; confidence interval, Δ*x* = ±0.26 %) and of retention time (RSD = 0.53 %, *p* < 0.05, Δ*x* = ±0.015 min) were determined. Interday precision (intermediate precision) was evaluated, as well. Of the samples, 20 μL of the analyte were examined within three consecutive days (*n* = 3 for each day). It was found that RSD of the peak area measurements was = 0.43 % (*p* < 0.05, Δ*x* = ±0.33 %) and of retention time 4.86 %, *p* < 0.05, Δ*x* = ±0.124 min.

Stability of the solution of (−)-*trans*-paroxetine hydrochloride was examined within seven consecutive days (0, 24, 48, and 168 h, *n* = 2 for each sampling time). RSD of the results of peak area and retention time measurements, respectively, were calculated: 0.48 %, *p* < 0.05, Δ*x* = ±0.41 % and 9.2 %, *p* < 0.05, Δ*x* = ±0.253 min.

### Comparative study concerning the developed method and already published one [9]

Both methods for the separation and determination of (−)- and (+)-*trans*-paroxetine were carefully validated. With use of ovomucoid stationary phase resolution (*R*_*s*_) was 2.60 (mobile phase, 10 mM phosphate buffer pH 3.5-acetonitrile 98:2, *v*/*v*, *t* = 23 °C, flow rate 1.5 mL/min)^1^ and on tris(3,5-dimethylphenyl)carbamate stationary phase-1.5 (mobile phase, hexane-ethanol-ethanolamine (80:20:0.2, *v*/*v*/*v*); flow rate, 1.0 mL/min; *t* = 23 °C; this result was not in agreement with previously reported [9], where *R*_*s*_ = 2.78 was claimed, but in the present study modified mobile phase was used). The elution order of the enatiomers was (−)- before (+)-enantiomer on ovomucoid, but (+)- before (−)-enantiomer on amylose tris(3,5-dimethylphenyl) carbamate stationary phase (Electronic supplementary material (ESM) Fig. S1). Both (−)- and (+)-*trans*-paroxetine exhibited linear response in the range of 0.01 to 100 μg/ml (*R* ^2^ ≥ 0.998).

**Fig. 2:**
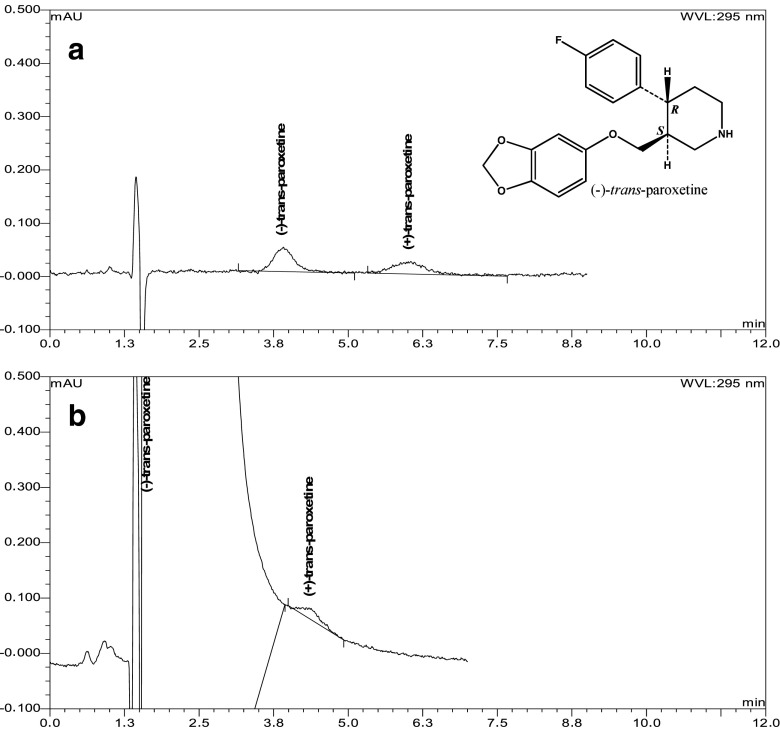
Chromatograms representing: **a** LOQ for (−)- and (+)-paroxetine enantiomers and **b** (−)-paroxetine containing 0.01 % (+)-paroxetine. Chromatographic conditions: silica-bound ovomucoid (Ultron ES-OVM 150 × 4.6 mm, 5 μm) stationary phase; mobile phase: 10 mM phosphate buffer pH 3.5-acetonitrile 98:2, *v*/*v*, *t* = 23 °C, flow rate 1.5 mL/min

Both limit of detection, LOD, and limit of quantification, LOQ (estimated as the amounts for which signal-to-noise ratios were S/N > 3 and > 10, respectively), were significantly lower on ovomucoid (LOD 0.005 and 0.006 μg/mL and LOQ 0.016 and 0.020 μg/mL for (−)- and (+)-paroxetine, respectively; Fig. 2a) than on amylose tris(3,5-dimethylphenyl)carbamate stationary phase (LOD (0.020 μg/mL) and LOQ (0.060 μg/mL) for (−)- and (+)-paroxetine). Even 0.01 % of (+)-paroxetine can be detected in the presence of (−)-paroxetine on ovoomucoid stationary phase (Fig. 2b). Absence of excipients peaks within analytical retention times proves good selectivity of the developed procedure.

### Determination of (−)-trans-paroxetine in medicinal products (ParoGen- and Xetanor-coated tablets)

**Fig. 3:**
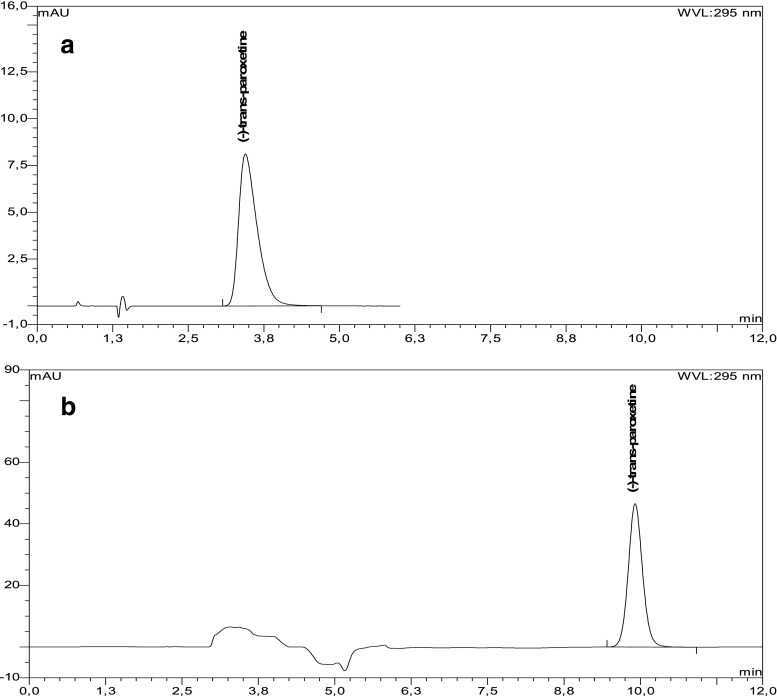
Chromatograms of ParoGen 20 mg coated tablets on **a** silica-bound ovomucoid (Ultron ES-OVM 150 × 4.6 mm, 5 μm; mobile phase: 10 mM phosphate buffer pH 3.5-acetonitrile 98:2, *v*/*v*, *t* = 23 °C, flow rate 1.5 mL/min) and **b** amylose tris(3,5-dimethylphenyl)carbamate (Chiralpak AD-H 250 × 4.6 mm, 5 μm; mobile phase: hexane-ethanol-ethanolamine 80:20:0.2, *v*/*v*/*v*, flow rate 1.0 mL/min, *t* = 23 °C)

(−)-*trans*-Paroxetine contents were determined in ParoGen- and Xetanor-coated tablets with the aid of ovomucoid as well as amylose carbamate stationary phases (Fig. 3, Table 1). They were found to be between 21.13 and 22.27 mg, with RSD between 0.67 and 1.14 %. Recovery of the analyte was in the range of 98.35–101.87 %. Thanks to suitably low LOQ (16 ng/ml), the method possibly could be recommended for the determination of paroxetine enantiomers in body fluids.

**Table 1:** (−)-*trans*-paroxetine determination in Parogen- and Xetanor-coated tablets

	Ovomucoid		Amylose tris(3,5-dimethylphenyl)carbamate	
	ParoGen	Xetanor	ParoGen	Xetanor
Content [mg]^a^	21.47 ± 0.18	22.27 ± 0.16	21.13 ± 0.25	22.17 ± 0.16
RSD (content), %	0.81	0.70	1.14	0.67
Recovery, %^b^	101.87	100.34	99.97	98.35
RSD (recovery), %	0.89	0.61	0.87	0.41

Confidence level 0.05

^a^ *n* = 6

^b^ *n* = 9

Purity of the examined formulations was also checked. According to European Pharmacopeia 7.7 monograph for active substance “Paroxetine hydrochloride anhydrous”, an amount of D contamination, (+)-*trans*-paroxetine hydrochloride, should not exceed 0.2 % with respect to (−)-*trans*-paroxetine hydrochloride. In the examined tablets, no (+)-*trans*-paroxetine enantiomer in the amount ≥0.01 % was found under available analysis conditions.

## Conclusion

New chromatographic method for the enantiodifferentiation of (±)-*trans*-paroxetine and the determination of (−)- and (+)-*trans*-paroxetine contents was developed with the aid of ovomucoid stationary phase. The new method is selective, precise, accurate, and offers good recovery (>98 %). Resolution, *R*_*s*_ = 2.8 is similar to the previously reported [10], 2.78. However, it is faster and few times more sensitive (LOD and LOQ, 5 and 16 ng/mL, respectively, comparing to 20 and 60 ng/mL, obtained for modified procedure [9]). The obtained results (elution order) shows that (+)-*trans*-paroxetine (as compared to (−)-enantiomer) forms more stable solute-stationary phase complex on ovomucoid than on amylose tris(3,5-dimethylphenyl)carbamate stationary phase. The developed method allows for the determination of active substance paroxetine hydrochloride in bulk and in pharmaceutical formulations according to European Pharmacopeia 7.7 requirements [14]. In the examined pharmaceutical formulations, ParoGen- and Xetanor-coated tablets, no (+)-*trans*-paroxetine enantiomer in the amount ≥0.01 % was found under analysis conditions.

## Electronic supplementary material

Below is the link to the electronic supplementary material.

ESM 1 PDF 280 kb

## Footnotes

1
*R*_*s*_ = 2.82 for flow rate 1.0 mL/min.
