# Supplementary material for: New validated HPLC methodology for the determination of (−)-trans-paroxetine and its enantiomer in pharmaceutical formulations with use of ovomucoid chiral stationary phase
Source: Anal Bioanal Chem. 2014 Jan 10;406(15):3697–702. doi: 10.1007/s00216-013-7565-y (PMC4026622; doi:10.1007/s00216-013-7565-y)
Supplement: Supplementary file 1 — PDF 280 kb [file 216_2013_7565_MOESM1_ESM.pdf]

Analytical and Bioanalytical Chemistry

Electronic Supplementary Material

New validated HPLC methodology for the determination of (-)-*trans*-paroxetine and its enantiomer in pharmaceutical formulations with use of ovomucoid chiral stationary phase

Małgorzata Lisowska-Kuźmicz, Małgorzata Kantor-Boruta, Anna Jończyk, Małgorzata Jarończyk, Agnieszka Ocios-Bębenek, Aleksander P. Mazurek, Zdzisław Chilmonczyk, Maciej Jarosz

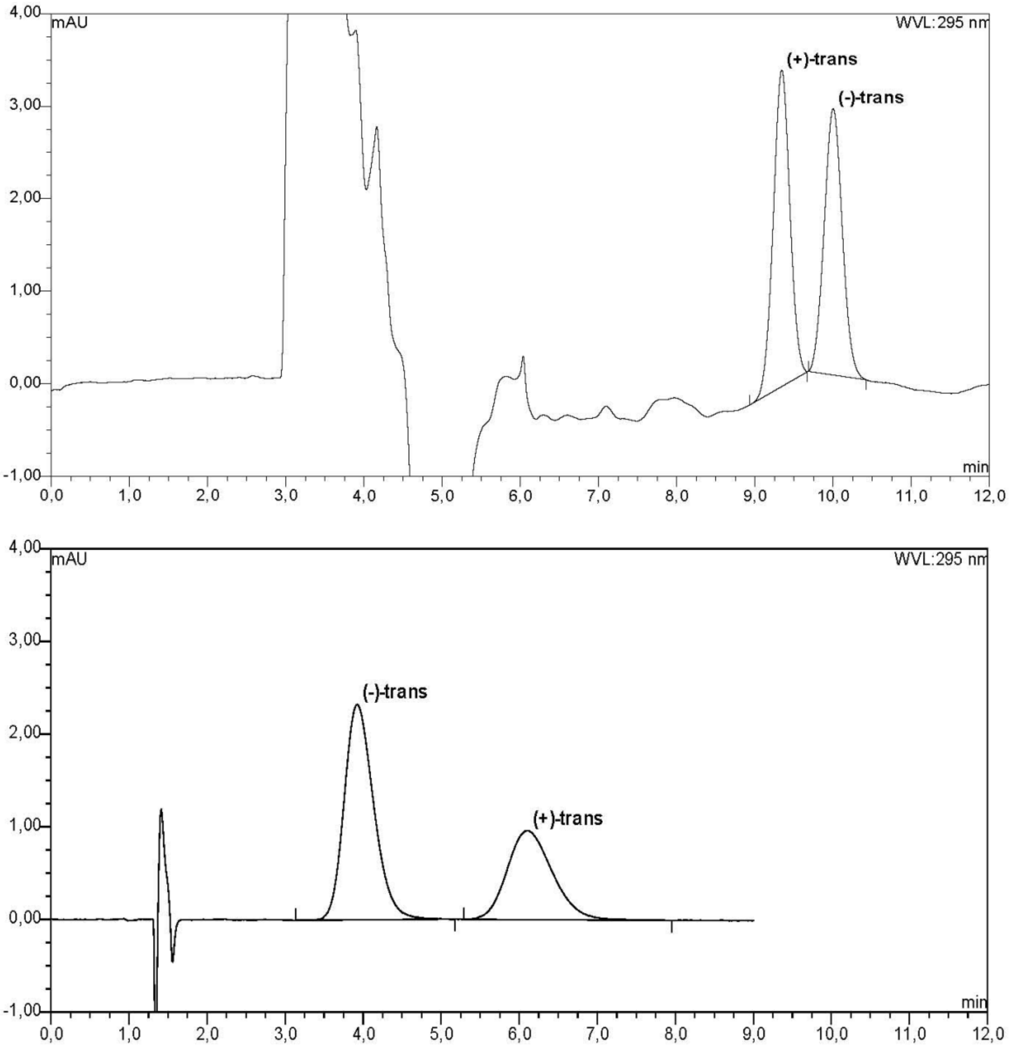

Fig. S1. Resolution of *trans*-paroxetine enantiomers on stationary phases: amylose tris(3,5-dimethylphenyl)carbamate (Chiralpak AD-H 250 x 4,6 mm, 5μm) (top) and silica bound ovomucoid (Ultron ES-OVM 150 x 4.6mm, 5μm) (bottom)
